# Management and Monitoring of IoT Devices Using Blockchain †

**DOI:** 10.3390/s19040856

**Published:** 2019-02-19

**Authors:** Kristián Košťál, Pavol Helebrandt, Matej Belluš, Michal Ries, Ivan Kotuliak

**Affiliations:** Faculty of Informatics and Information Technologies, Slovak University of Technology in Bratislava, 842 16 Bratislava, Slovakia; kristian.kostal@stuba.sk (K.K.); xbellus@stuba.sk (M.B.); michal.ries@stuba.sk (M.R.); ivan.kotuliak@stuba.sk (I.K.)

**Keywords:** IoT, blockchain, management, security, monitoring, Hyperledger Composer

## Abstract

Nowadays, we are surrounded by a large number of IoT (Internet of Things) devices and sensors. These devices are designed to make life easier and more comfortable. Blockchain technology, especially its mass application, is becoming a term number one. Adoption of blockchain into enterprise networks still has a few challenges that need to be tackled. Utilizing blockchain can bring increased security and efficiency of network maintenance. The key feature of the blockchain, immutability, brings resistance to unauthorized modifications. The whole history of device configuration changes is stored in the blockchain, hence recovery after incidents is very straightforward. This paper extends our previous studies. We are introducing an improved architecture for management and monitoring of IoT devices using a private blockchain. The majority of the system is built on a chaincode, which handles CRUD (Create, Read, Update, Delete) operations as well as encryption and access control. Device configuration files are stored in the blockchain. When a modification occurs, the device downloads a new configuration in a simple manner. The chaincode receives notification whether setup was successful and this history is available for administrators. Our results show that such a system is possible and dissemination of configuration changes to IoT devices can be secured by the blockchain. The key novelty of our solution is a distributed management of configuration files of IoT devices in enterprise networks utilizing blockchain technology. This is essentially improving security and storage options for configurations in the blockchain.

## 1. Introduction

A blockchain, also called a distributed shared ledger, is an immutable database of records secured by cryptography. It allows exchange and storage of digital assets without the need for third party oversight. Devices that download a configuration file from a centralized server need to trust that authority and if it is compromised the device becomes vulnerable. With a blockchain, the need for central authority is absent. Devices exchange assets directly between each other—peer-to-peer.

The blockchain offers some key properties that distinguish it from a generic database. Firstly, it is distributed by default. If someone wants to run a blockchain network with only one node, it is pointless. Next, records in the blockchain are immutable, which means one cannot delete or modify them. Doing so, it would break the validity. Records can be only updated by adding a completely new record. In this way, a history of all changes is securely kept. The blockchain uses smart contracts (chaincode) to ensure a correct execution of predefined business rules. A smart contract is a computer program, or in other words an agreement, that is run by nodes in the blockchain for automation. We also use smart contracts for management of configuration files in an Internet of Things (IoT) environment.

Internet of Things is in information technology a designation for connection between built-in devices and internet. The connection of devices should be wireless and should bring new possibilities for interactions between systems and also bring new options for their control, tracking and introduction of advanced service capabilities.

IoT devices are limited by design, with constraints on available resources and performance compared to the network devices in enterprise environments. This has to be taken into consideration when designing management and monitoring system for IoT devices. Heterogeneity of IoT devices for various purposes uses different configurations and provides different levels of monitoring. Similar heterogeneity of devices in enterprise network environments enables adaptation of existing network monitoring and management systems for IoT deployment.

Enterprise networks and public access networks, as a communication backbone within organizations or communities, are a widely spread communication enabler across the world. Due to the constant need for a better, more secure connectivity of all nodes, the whole sector of enterprise networks is in systematic development and open to innovations. The key innovation should focus on the following key aspects: increasing network performance, increasing security and reducing maintenance costs by application of blockchain technology. We have already presented our initial results for enterprise networks in a conference paper [1].

At the same time, requirements for securing network access using blockchain are a subject for a separate research. These requirements depend on many factors determined mainly by the characteristics of the network, e.g., by its topology. Nonetheless, network monitoring forms an important database used for security control.

We define our hypothesis that a blockchain can be used as a platform for management of IoT devices in practice. The word management incorporates handling configuration files for IoT devices; these files can have various formats, which are device specific. We believe a blockchain can be used to achieve enterprise-level security, create trust and resolve potential issues among different parties, mainly in the enterprise sector.

Monitoring and management of enterprise networks are important, even essential issues for reliable provisioning of services. There are multiple approaches to monitoring the status of network nodes and measuring their performance. Necessary changes to the configuration of network nodes can be made automatically based on the observation of the network behavior. Most of the popular network monitoring systems and tools [2] focus primarily on measurements and tracking of network status. They usually provide various levels of management through plugins and extensions. One approach to network monitoring is the active monitoring utilizing probes for measurement of network performance metrics. Pairs of network devices can be configured to inject additional traffic into a network to monitor key metrics, e.g., one-way or round-trip latency. Service Level Assurance, more commonly known as IP SLA, is an implementation of active network monitoring originally introduced by Cisco with the draft for inter-vendor compatibility outlined in IETF Request For Comments (RFC) 6812 [3]. Other active network measurement approaches are IETF One-way Active Measurement Protocol (OWAMP) [4] and Two-Way Active Measurement Protocol (TWAMP) [5]. The main disadvantage of active monitoring is additional traffic and its impact on available resources. However, passive monitoring of metrics, such as round-trip latency, is not possible.

In contrast, passive network monitoring does not introduce additional traffic and only collects existing data or metadata about the traffic already in the network. Examples collecting a copy of all data are known as port mirroring, with user traffic being copied and sent to a server with a sniffer software for collection and analysis. However, monitoring all traffic in this way would in effect require separate monitoring network with the same parameters and resources as the monitored network. Accordingly, only a small portion of the traffic in an enterprise network is usually mirrored, with only metadata about traffic flows collected for all traffic. NetFlow and IP Flow Information Export [6] are used for a passive collection of traffic metadata.

Monitoring of network performance and traffic presumes functioning network devices and their interconnection. Device and interface statuses need to be tracked for gaining a comprehensive overview of network conditions. The accepted method for recording changes of status information is Syslog with corresponding protocol [7] for transfer of notification messages over the network. While Syslog is used in general for tracking changes on any PC, server or device, Simple Network Management Protocol (SNMP) is a specialized protocol for monitoring and basic management of network devices.

All of the mentioned methods have advantages and disadvantages. Using a combination of them with different settings for different situations and goals is necessary. This point is argued in [8] and together with methods to minimize the impact on network performance by the positioning of active probes and passive monitoring devices in [9]. Applications and modifications for active and passive network monitoring methods possible in Software-defined networking (SDN) are explored in OpenNetMon [10].

Information about networks accumulated from multiple sources is consolidated, analyzed and evaluated in Network Monitoring System (NMS) applications which simplify and can also automate network management. Popular open source NMS tools are Zabbix and Nagios as well as the abundance of proprietary network monitoring and management suites from various device vendors.

Management of network devices in enterprise networks is accomplished in multiple ways. With direct administration through Command-Line Interface (CLI) being almost universal but also most manually labor intensive and as such does not scale well. Managed networks of increasing size and complexities require more systematic and automated solutions. NETCONF [11] and SDN [12] are showing promise of increased automation. SNMPv3 added provisions for remote device configuration. However, SNMP is still primarily used only for monitoring because support for modification of device attributes is very dependent on vendor implementation. On the other hand, NETCONF is focused on device configuration through open Application Programming Interface (API) using eXtensible Markup Language (XML) based models of device behavior.

Generally, all blockchain systems can be divided into two categories: public and private. A public blockchain will consist of multiple nodes connected in a decentralized way while anyone can join or leave the network. This creates more challenges for the system design as any of the connected nodes may behave maliciously in order to manipulate assets defined on the blockchain. A private blockchain is usually set up between parties that want to exchange certain digital assets with only authorized nodes allowed to participate in the network consensus. Any request from a foreign node is discarded. An experiment in [13] showed that Hyperledger performed better in transactions per second as compared to the Ethereum private network. This is mainly due to different consensus algorithms [14,15]. Integration of blockchain and IoT is an area of considerable research opportunities.

The most studied approach is the utilization of blockchain and smart contracts together with IoT sensors in the product supply chain management and its optimization. Kshetri in [16] explored real world use-cases, where blockchain and IoT sensors were utilized in supply chains of food, pharmaceutical and other industries for traceability of raw resources, ingredients or parts. They note that, while there are some legislative and technological challenges, scalability and flexibility of blockchain can increase the security of supply chains and streamline their management. Saberi et. al. in [17] explore and categorize barriers in the adoption of blockchain technology in supply chain. Improving supply chains with the help of IoT is examined by Woo et. al. [18] to supply information for big data analytics and modeling, and Qu et. al. [19] for process synchronization and real-time logistics.

Enhancement of supply chain management with IoT and blockchain ideas can be applied to utilities like distribution and metering. Smart homes and smart communities are one such application of IoT sensors and blockchain, where Alcarria et. al. [20] proposed the architecture for enabling trustworthy resource monitoring of utilities for all members of a smart community. Members of such smart communities can better monitor and optimize consumption of resources in a secure and transparent manner.

A different way of utilizing blockchain is to augment the IoT infrastructure to increase its scalability and optimize its management. Devices in an IoT environment can be considered similar to network devices with respect to their management and monitoring, but there are key differences. Firstly, due to their relative simplicity and long-term deployment with limited resources, IoT device bandwidth is usually constrained and the device may not be always online to conserve those limited resources. Data integrity and security are named by Panarello et. al. in [21] as main challenges of IoT that can be improved by the introduction of a blockchain.

One approach to disseminating software updates in an IoT environment with the utilization of a blockchain is PatchTransporter [22]. IoT devices transport patches among themselves in a P2P manner. The process is done with a blockchain based smart contract used for validating transportation of correct patches and to provide an incentive. The incentive is considered for self-interested devices to participate in the distribution of patches to other devices.

Private [23] blockchain can be used in a network management as a timely and secure method to disseminate configuration changes to the nodes. Compared to the more centralized traditional approach to network management, the distributed nature of blockchain allows for greater scalability and increases reliability and availability by eliminating a single point of failure. Additionally, once configuration changes are written into a blockchain, there is no possibility of a record being tampered with. In the event of misconfiguration, rollback to the previous working configuration is straightforward [24].

We propose a new network monitoring and management architecture based on blockchain technology. Network administrators control network devices indirectly by recording modification of device configuration into blockchain that network devices check for updates to their configuration. This contrasts with a direct configuration of devices through CLI in more traditionally managed networks.

In this section, we provided an analysis and overview of the current state-of-the-art technologies. The rest of this paper is structured as follows. In Section 2, we describe original architecture design for management of configuration files in enterprise networks. Section 3 introduces improved architecture design for the IoT environment. Our results are shown in Section 4 and further discussed in Section 5. Materials and methods used in our solution are described in Section 6 supplemented by the source code published on GitHub. Conclusions, future work, and additional improvements are explained in Section 7.

## 2. Design

**Figure 1:**
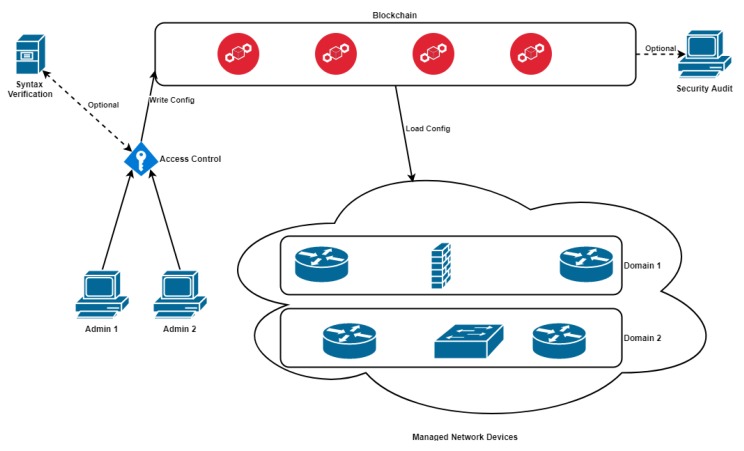
Proposed architecture for network monitoring and management over blockchain.

The whole architecture is shown in Figure 1. Authorized network administrators use digital certificates to authenticate themselves, and then they can modify the configuration of devices recorded in the blockchain, given they are authorized to do so for a given device or group/domain of devices. Authentication certificates can be managed by utilizing blockchain-based framework described in [25] as an alternative to standard Public Key Infrastructure (PKI). The correctness of the new configuration should be validated by a syntax verification to minimize the chances of accidental human errors introduction into configuration recorded in the blockchain. The administrator’s certificate is also used to sign the new configuration for identification and attribution purposes. The transaction consists of a timestamp, admin ID, device ID, and the encrypted device configuration. Once the transaction is written to a new block and added to the blockchain, the information is distributed to blockchain network peers. The addition of a new block to the blockchain triggers event informing all managed devices of a new addition so it can be checked by a device ID as to whether the change affects its configuration. The device then downloads the encrypted configuration from blockchain, using its private key for decryption, and applies the modifications. History of all the changes is available in the blockchain for review by security and audit teams.

**Figure 2:**
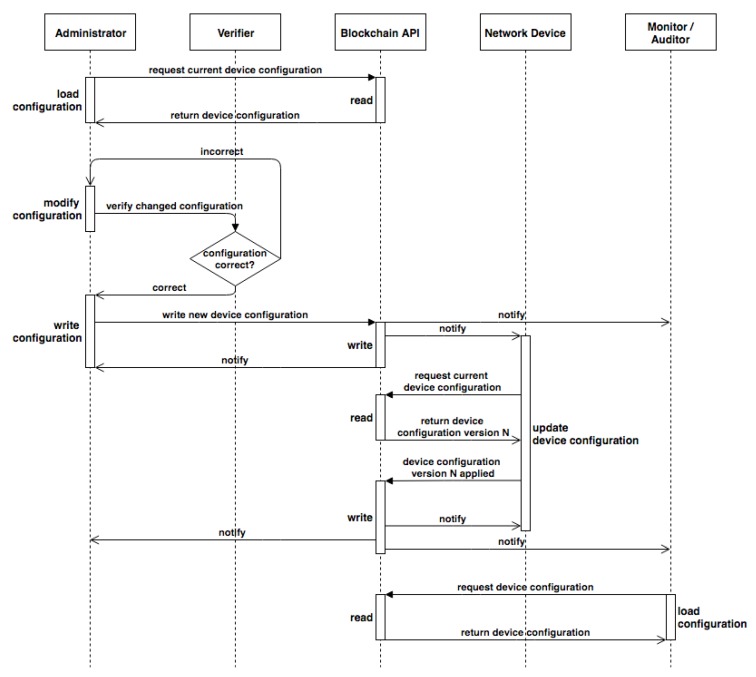
Management and monitoring sequence diagram.

The process flow is outlined as follows (see Figure 2):

1. Admin $A_{x}$ ($A_{1}$, $A_{a}$) loads config for device $D_{y}$ ($D_{1}$, $D_{n}$) or group of devices $G_{z}$ ($G_{1}$, $G_{m}$) from the blockchain and decrypts it using admin key $AS_{x}$.
2. Ax modifies the configuration.
3. New configuration is sent for verification to have syntax validated to minimize human errors.
4. Verified configuration is encrypted and recorded in a new block together with admin ID, device ID, and timestamp. The block is added to the blockchain.
5. Participants are notified about a new block.
6. Affected device $D_{y}$ downloads the block, decrypts configuration and applies changes.
7. Device $D_{y}$ adds a new block into the blockchain with information about whether the new configuration has been applied successfully, together with the hash of the configuration and timestamps of download and application.

### 2.1. Proof of Concept

As a blockchain implementation, we have chosen to use Hyperledger Composer [26], mainly because of the way business networks are implemented. Hyperledger Composer also contains a tool to automatically generate Representational State Transfer (REST) API according to the business model. This API is used by the modified Trivial File Transfer Protocol (TFTP) server to query data from the blockchain.

### 2.2. Modified TFTP Server

Furthermore, we use Cisco routers, as a representation of a network device. Routers are initially configured to connect to a TFTP [27] server in a periodic interval and download and apply their configurations. While we investigate other TCL options (connecting to REST API directly from TCL script), this remains our temporary workaround. In order to get a configuration from a blockchain to a router, the router will connect to the TFTP server which will provide it. Hence, a modification to the TFTP server is needed. When TFTP receives a read request from a client, it looks up in files in its working directory. If there is a file with the requested name found, the TFTP server sends its content to the client. Instead of looking for the file on a disk, our modification will query Hyperledger’s REST API for the requested configuration. If it is found, its content is temporarily stored in TFTP’s working directory and served as a normal file. This way, the router is believed that it got the configuration from the TFTP server, but the configuration really came from blockchain.

### 2.3. Hyperledger Composer Business Network Definition

Currently, the business network is defined in a very simple fashion, one asset called Config which contains an ID and the configuration data itself. Data are represented as an array of strings, one index for each line in a configuration file. The definition of such asset in Hyperledger Composer can be seen in the following code snippet:
namespace sk. fiit .routernetwork  asset Config identified by configId {o String configIdo String[] data}
Participants, access control and transaction definitions are left blank for now.

## 3. Improved Design

**Figure 3:**
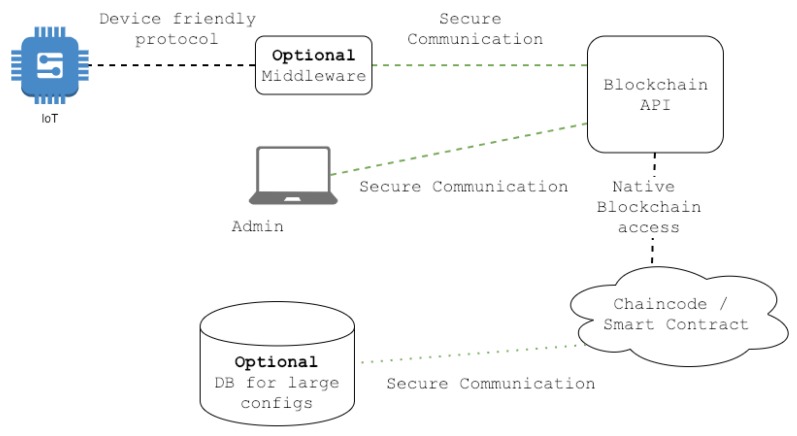
Improved architecture.

With a few modifications, the outlined design can be also used in IoT devices. Since security is an important part of IoT, we focus on improving the security model. The new architecture is shown in Figure 3. IoT devices are usually capable of sending HTTPS requests; therefore, middleware is just an optional node that can be used when certain IoT devices cannot support HTTPS. Another noticeable node is the off-chain database that is also optional just like middleware. Since configuration files for IoT devices are usually small (less than few megabytes), the blockchain should have no issues handling them. However, if the administrator decides that they will use the external database, the option is there. We do not recommend using the off-chain database because it breaks the principles of blockchains and distributed systems. Chaincode is the most important part of our system; it handles authentication, authorization, cryptography, and access to the blockchain. Syntax verification, access control, and security audit are now parts of the chaincode as well. In the previous design, these modules were thought to be standalone, which is still possible.

### 3.1. Cryptography

The main design principle is that chaincode cannot hold any cryptographic keys. All necessary keys are only passed as arguments. The identity of a user is a pair consisting of a username and password. A correct identity plus a key must be supplied to the chaincode to perform one of the CRUD operations. If correct login and a wrong key will be supplied, read, update and delete will fail. Since the chaincode will try to decrypt the config as a security measure if the decryption fails, the whole request fails. This is what we call Key Verification Routine. Create is a special operation because the config does not exist yet. There is no way to verify if the key is valid, hence the config is encrypted with the provided key and stored. If this was a forged request, an attacker must still modify the network device such that it downloads the config with the obtained id. It is assumed that the attackers do not have an access to modify the network device configuration. If they have, it is a security issue out of our scope. Hence, the worst-case scenario is an arbitrary config sitting in the blockchain or a database that will never be accessed by a network device.

### 3.2. Remote Database

With the improved design, we add the ability to use an external database as a storage for large configuration files. If there is a need for an off-chain database for some reasons, chaincode can connect to the off-chain database, but the credentials and IP address of that database must be supplied. Accessing a centralized system via a decentralized one is not recommended because the data stored in the off-chain database are not protected by the blockchain and anyone with the access to the database can modify them. There are various options when it comes to off-chain solutions; to mention a few: a file system; another blockchain; NoSql; SQL; TFTP; SFTP.

### 3.3. CRUD Operations

CRUD (Create, Read, Update, Delete) operations are designed with security in the first place. These operations are implemented using Hyperledger Composer. The requests are sent to Hyperledger Composer REST API, which then forwards them to the chaincode, where the actual code is executed.

Create operation starts with the chaincode receiving a request to create a new configuration. It contains fields such as login, password, encryption key and the configuration data itself. The user (identified by login and password) is authenticated and then authorized against an ACL. If authentication and authorization were successful, the configuration is encrypted and stored to a blockchain or a remote database, based on settings. A hash of configuration data (plain text) is also calculated and stored to the blockchain. The administrator, who initialized create operation is notified of the result and obtains the configuration ID. Create operation is logged to the blockchain.

Read request contains fields such as login, password, decryption key and configuration ID. Authentication and authorization are performed just like in create operation. If successful, a key verification routine is performed. The key verification routine tries to decrypt the configuration file using the provided key. Once decrypted, the stored hash is compared to the calculated hash. If the hashes are equal, the key verification routine was successful and decrypted, the configuration file is sent to an administrator or a device—depending on who initiated the request. If the request came from an IoT device, a message regarding the status of the application is sent to the blockchain. This action is logged.

Update operation is much like read operation, with few changes. The request contains arguments such as login, password, decryption key, original configuration ID and a new configuration file. After authentication and authorization succeed, the key verification routine is performed. If successful, configuration data stored in the blockchain are updated with the new configuration data and a new hash value is calculated and stored to the blockchain. This action is logged and the administrator is notified of the result.

Arguments for delete operation are login, password, decryption key and configuration ID. As with other operations, authentication, authorization and key verification routine are performed. If successful, configuration is marked as deleted and no further operation is allowed. Subsequent read, update or delete operations result in a same way as if there is no configuration stored under given ID. This action is also logged and admin is notified of the results from the delete operation.

### 3.4. Known Limitations

When the encryption key is compromised, related configuration files must be re-encrypted with a new key. However, old configurations can be still theoretically accessed because of a blockchain append-like system. A potential security issue may arise when the attacker decrypts an old configuration, stored in a block that was created in the time when the compromised key was still valid.

## 4. Results

To verify our solution, we have decided to implement a prototype of configurations management system in a Cisco environment. The universality of the architecture for IoT environment was tested in two scenarios. Firstly, a functional scenario was used to verify the utility of the proposed design; secondly, a scenario was used to measure times needed to download, accept and install an updated configuration file. Both scenarios were validated in a certified Cisco Lab [28] at our university. We used Cisco routers that can be described as any IoT device or sensor which has some functionality and configuration. For use in a Cisco environment, we have created two TCL scripts for the router—one script for downloading configuration and one script for uploading configuration to the TFTP server. The upload script is used for development purposes only. The download script is set up to run every two minutes (in CRON terminology every even minute). The script connects to the TFTP server and obtains the requested configuration. Although there are other options besides TFTP, e.g., SSH or standard FTP, we decided to use TFTP in our prototype for its simplicity.

A prototype testing topology was configured to contain only two physical devices directly connected by the Ethernet. One was Cisco 2811 router running IOS 15 representing an IoT network device to be configured through a blockchain. The modified TFTP server for a connection to the Hyperledger Composer based blockchain was implemented in Python and ran on Mac OS X High Sierra. The goal of our first test scenario was to successfully obtain device configuration via modified TFTP server that served as an intermediator to Hyperledger Composer REST API. We have tested manual and automatic (CRON like) invocation of the download script and both tests yielded successful results. The new configuration was modified via composer–playground User Interface (UI) and applied within two minutes of modification on the target device.

The second scenario consisted of 10 measurements of time used to download and install an updated configuration file onto the Cisco router. Manual invocation of the download script was set up for this scenario. The size of TCL script was 1.15 KB, the size of the new configuration file was 2.87 KB and the link speed between devices was 100 Mbps. Results from ten different measurements of time needed to download and update the configuration on the Cisco device are shown in Table 1.

## 5. Discussion

The results shown in the previous section prove that it is possible to use the blockchain network for management of configuration files in an IoT ecosystem. Any IoT device connected into our blockchain architecture can be managed and monitored. If it needs a new configuration or settings, a verified user inserts them into the blockchain, from which the IoT device directly or optionally with help from middleware (e.g., TFTP server) downloads new information and notifies about a successful or unsuccessful setup. This means that administration of an IoT network can be decentralized, distributed, immutable and provenances of all configurations are known. These characteristics are achieved through the utilization of blockchain technology.

The first test scenario directly supports the hypothesis of using blockchain network for management and monitoring of IoT devices. It further proves our architecture design and its utility. The second scenario was aimed at performance and time measurement. Looking at the results, the average total time needed for the device to download and update configuration is 402.8 ms. The median of the same total time is 402.5 ms. Considering these numbers, we can claim that the configuration is updated in a nearly seamless way and the time below 0.5 s is not noticeable. Note that we have measured mentioned times on Cisco routers with enough computational power so the results should be perceived as illustrative, and, on small IoT devices with just enough performance to work, could be different.

## 6. Materials and Methods

Our source code is available at GitHub [29]. This repository includes Cisco TCL scripts for upload and download of configuration from TFTP server. Source code for a simple TFTP server is also available. It is modified to connect to a Hyperledger Composer REST API, request the configuration from there and then create a regular file that is served and transferred according to TFTP specification. The IP address in the TFTP server points to the virtual machine, where Hyperledger Composer runs. The Hyperledger Composer network is started using the Vagrant box; note that VirtualBox must also be installed [30]. Follow the readme file in the Vagrant machine.

## 7. Conclusions and Future Work

As discussed in this article, the IoT environment provides many challenges compared to enterprise environments. IoT devices are not always online, whereas enterprise network devices are. Furthermore, available resources such as bandwidth, battery life, CPU performance, and instruction set are limited. Support for already low-level cryptographic functions and primitives (e.g., SHA) on IoT devices is often minor or even missing completely, resulting in much greater time for executing cryptography related routines. To overcome these challenges, we modified our original architecture. With respect to the original paper [1], we improved our architecture and added a much needed security model. The new architecture is fully decentralized and does not depend on any centralized node. For the security concept of our solution, we have added verification of a configuration file integrity. Using secured communication between nodes, we are also successfully maintaining the confidentiality of messages. To further prevent unwanted reads by other blockchain participants, a cryptographic function is used in chaincode. The chaincode does not hold any decryption keys; they are only passed as arguments of API calls. Finally, we have implemented access control for users, admins and also devices, so there is only a little possibility left that someone can gain unauthorized access to our system.

Our solution provides a platform for management of IoT devices configurations with the ability to verify the configuration before deployment and irrefutable history of all changes through blockchain. On the other hand, Lee’s PatchTransporter [22] utilizes blockchain only to incentivize IoT devices to spread patches among themselves directly in a peer-to-peer network. The key novelty of our solution is distributed management of configuration files of IoT devices in enterprise networks utilizing blockchain in a secure way. This is essentially improving security and storage options for configurations in the blockchain. We have evaluated our proposal and reviewed the results in Section 5.

However, additional modifications would be appropriate for future work to enhance our architecture, at least the following improvements stated below.

### 7.1. Extension to Use Blockchain Based PKI

To further integrate blockchain solutions, a blockchain based PKI such as [25] can be integrated with our system instead of traditional PKI based on centralized certification authorities.

### 7.2. Emission of Notification That a New Configuration of a Device Is Available—Integrated Message Queue

Instead of periodic checking of configuration updates, a more sophisticated system can be used. A streaming message queue application such as Apache Kafka can be integrated to help with the update notifications. Whereas IoT devices are not always online, a message queue system can hold update or other messages until the IoT device wakes up and pulls the messages.

### 7.3. Extended Monitoring Capabilities

Currently, we mean by monitoring the ability to check which configuration is running. For future work, it might be useful to know more information about the device, such as battery level, CPU utilization, RAM and disk space used. To achieve this, low-level monitoring, such as Zabbix or Nagios, can be integrated.

### 7.4. Support Public Blockchains Like Ethereum

We have chosen Hyperledger Composer as our platform for blockchain because it provides the functionality we need while minimizing the time needed for implementation and setup. This does not mean that our system can not be implemented using other blockchain platforms such as Ethereum.

## Figures and Tables

**Table 1:** Measurement of time needed to obtain and install updated configuration file into Cisco router.

Nr. of Measurement	Download (ms)	Setup (ms)	Total Time (ms)
1	9	394	403
2	10	392	402
3	9	391	400
4	11	404	415
5	11	396	407
6	10	395	405
7	8	399	407
8	11	377	388
9	10	389	399
10	12	390	402
